# A State of the Art of the Overall Energy Efficiency of Wood Buildings—An Overview and Future Possibilities

**DOI:** 10.3390/ma14081848

**Published:** 2021-04-08

**Authors:** Matheus Roberto Cabral, Pierre Blanchet

**Affiliations:** NSERC Industrial Research Chair on Ecoresponsible Wood Construction (CIRCERB), Forest and Wood Sciences Department, Université Laval, 2425 rue de la Terrasse, Quebec City, QC G1V 0A6, Canada; pierre.blanchet@sbf.ulaval.ca

**Keywords:** construction, energy efficiency, efficiency, embodied energy, mass timber, materials, phase-changing materials, post-and-beam, wood composites, wood-frame

## Abstract

The main goal of this study was to review current studies on the state of the art of wood constructions with a particular focus on energy efficiency, which could serve as a valuable source of information for both industry and scholars. This review begins with an overview of the role of materials in wood buildings to improve energy performance, covering structural and insulation materials that have already been successfully used in the market for general applications over the years. Subsequently, studies of different wood building systems (i.e., wood-frame, post-and-beam, mass timber and hybrid constructions) and energy efficiency are discussed. This is followed by a brief introduction to strategies to increase the energy efficiency of constructions. Finally, remarks and future research opportunities for wood buildings are highlighted. Some general recommendations for developing more energy-efficient wood buildings are identified in the literature and discussed. There is a lack of emerging construction concepts for wood-frame and post-and-beam buildings and a lack of design codes and specifications for mass timber and hybrid buildings. From the perspective of the potential environmental benefits of these systems as a whole, and their effects on energy efficiency and embodied energy in constructions, there are barriers that need to be considered in the future.

## 1. Introduction

Due to increasing urbanization, cities have been forced to become larger and more complex. This leads to increased demand for housing, transportation, infrastructure, and energy systems. The combination of developing urban areas and the improvement of comfort parameters also has increased building energy consumption, making it one of the biggest concerns of today’s society [1,2]. According to the International Energy Agency (IEA), an increase of 2.3% in global energy consumption was reported in 2018, representing almost twice the average growth rate since 2010. Furthermore, the Organization for Economic Cooperation and Development (OECD) reports that due to higher energy consumption, global CO_2_ emissions rose to 33.1 Gt of CO_2_ (2019), an increase of 1.7% (2019) [2]. Among other aspects underlying this growth, it is widely recognized that more than 32% of total global energy expenditures and 19% of total greenhouse gas (GHG) emissions are attributable to energy processes in buildings, such as cooling and heating [3,4] In addition, building energy demand is expected to increase between 1.5% and 2.1% per year from 2012 to 2040 in OECD countries [5].

Therefore, given the impacts on and future perspectives of the energy sector, it is relevant for the construction industry to consider more energy-efficient buildings. Thus, many countries have developed their standards and certifications for buildings with lower environmental impact, including reduced energy consumption. For example, the rating and certification program Energy Star developed by the US Environmental Protection Agency (EPA) [6], the Leadership in Energy and Environmental Design (LEED) certification by the US Green Building Council (USGBC), the Building Research Establishment Environmental Assessment Method (BREEAM) by the Building Research Establishment (BRE) in the United Kingdom, the National Australian Built Environment Rating System (NABERS) by the government of Australia among others, which can be found in detail in the references [6,7,8,9].

Energy efficiency can be defined as approaches and technologies that demand less energy to produce the same quantity of services. In buildings, for example, energy is consumed directly from delivered energy sources, such as electricity and natural gas, which is commonly known as operational energy, and indirectly through the use of construction materials, known as embodied energy (EE) [10]. EE refers to the energy associated with the production of materials, i.e., the energy used to extract raw materials and process and manufacture materials, as necessary [11], as well as the energy used to transport materials to the site and assemble materials/components together [12,13,14]. Furthermore, for a better understanding, the difference between source energy and site energy must be clarified. Source energy refers to the total amount of raw fuel (or other power supply) that is required to operate the building. At the same time, site energy is the amount of heat and electricity consumed by the building, as reflected in your utility bills [15].

Thus, when considering the energy efficiency of a building, it is important to consider the EE of building materials in addition to operational energy. As several studies on EE and buildings show, the use of low-energy materials can reduce total EE by 25% to 60%, which subsequently leads to energy savings over the building’s life cycle [16,17,18,19,20,21,22,23,24]. For example, Stephan et al. [25] studied a hybrid input–output life cycle inventory for the energy demand of a passive suburban house in Belgium. The results showed that embodied, operational, and transport energy represented 40%, 33% and 27%, respectively, of the total energy used. Lessard et al. [26] showed that building life cycle analysis (LCA) results indicated the contribution of materials to environmental impact was >50% for the Quebec region in Canada, where more than 95% of the electricity is renewable. In addition, for the Ontario region (Canada), the contribution of energy consumption energy was 90% due to electricity generated from nuclear power. From the perspective of building energy efficiency, experts have stated that it falls into three broad categories: (i) building services, (ii) building design under specific weather conditions and (iii) building characteristics.

### 1.1. Building Services

Building services refer to heating, ventilation and air conditioning (HVAC) systems, including water heating, lighting and electrical systems, and control management. HVAC is a major concern since people spend about 80% of their time in buildings, according to Li et al. [27], and HVAC systems represent 47.7% and 51.0% of the energy consumed in residential and office buildings, respectively [28]. In North America, for example, about 40% of all energy consumed is used only for heating, as reported by the Canadian Wood Council [9]. Therefore, it is essential to find strategies to improve the energy efficiency of buildings. A strategy to reduce the impact of HVAC by using smart technologies and controls, as proposed by Stopps and Touchie [29], is relevant. Using thermally efficient materials to improve HVAC, such as radiant ceilings and thermally active building systems tested by Gärtner et al. [30] for flexible office spaces in Stuttgart (Germany), is another solution. Finally, applying methods, such as the Bayesian network technique to select the most energy-efficient primary HVAC systems, should also be considered [31]. Furthermore, according to the reference [32], another promising strategy for providing energy savings in buildings is the use of energy recovery systems. Mardiana-Idayu and Riffat [33] stated that an energy recovery system to building HVAC systems achieved the efficiency of close to 66% for sensible energy and the latent energy efficiency was nearly 59% gained.

### 1.2. Building Design under Specific Weather Conditions

The impact of weather on building design is affected by the construction conditions, such as outdoor air temperature, humidity, solar radiation and wind speed [34]. The heating demand of buildings is associated with the difference in temperature inside and outside; however, it is inversely proportional to solar radiation. Aspects such as increased air infiltration or the opening of windows can increase heat consumption, resulting in greater energy expenditures. In terms of energy efficiency, according to Aslani et al. [35], the envelope is one of the main elements of the building. They are the physical barrier between the external and internal environment of a building, providing resistance to air, heat, noise, light and water. Additionally, high winds generate higher outdoor convection coefficients, which increases heat loss by the envelope and infiltration [36]. In summer and very rainy conditions, i.e., when there were high temperatures and humidity, the type of material used in the envelope had a negative effect on indoor air quality comfort. Therefore, it is necessary to propose a design plan that factors in appropriate hygrothermal conditions to take energy savings into account [37]. The projection carried out by Berardi, and Jafarpur [3] for 2070 showed a considerable decrease (i.e., 18% to 33%) in heating energy demand as well as an increase of between 15% and 126% in cooling energy use, which can vary according to climatic usage and building typology. Another point to be considered in this topic is building daylight exposure. As pointed out by some studies, proper daylight is largely indicated as an important strategy to reduce building energy demand while also improving indoor environmental quality and the occupants’ productivity, satisfaction, and health [38,39,40,41].

### 1.3. Building Characteristics

This topic includes the building shape and the materials used to construct the system. The shape of a building influences the solar energy it receives and its total energy consumption. There is also a correlation between construction geometry, energy demand and glazing size in cold climate regions [42]. Some studies have pointed out that a building’s shape facilitates the transmission of heat losses in a cold climate and consequently increases the amount of energy required for heating. It is, therefore, crucial to find the optimal building shape with a minimal external surface. It is also important to mention that the glazing size and the building geometry directly impact the energy requirements of buildings, as pointed out in [43]. Building materials are an important factor in building energy performance. They usually include the insulation of the walls, roof, windows and foundation [44]. From the point of view of energy demand, the fabrication and operations (i.e., transportation) of building materials require an enormous amount of energy, not only in the construction phase but in all phases of their existence [45]. Furthermore, beyond the energy demand, it is estimated that building materials consume about 40% of all raw materials extracted and 25% of virgin wood [46]. In addition, construction is responsible for generating about 25% of all worldwide residues, with a projected increase from 79 Gt (2011) to 167 Gt (2060) [47]. There is, therefore, a huge need for the building sector to seek more sustainable development. To do so, construction must embrace a multidisciplinary approach with different features to improve building characteristics, which have been the subject of several studies. Some examples of feature studies have investigated materials with improved performance [48,49,50], reuse of water and materials [51,52,53], reduction of GHG emissions [54,55,56] and energy-efficient buildings [43,57,58,59].

Wood buildings have been widely constructed worldwide and account for 90% of single-family homes in North America, 45–70% in parts of Europe and 45% in Japan, respectively [60,61,62,63]. Moreover, it is quite well-known that wood buildings are lightweight and easy to build. In comparison to other construction methods, such as steel framing, concrete and masonry, wood buildings systems have better thermal performance because wood is a natural insulation material, not to mention the fact that wooden structures can be easily built and insulated, which consequently leads to energy savings [64,65].

Therefore, taking into account that materials can have a significant impact on the energy efficiency of a building, the main objective of this article is to present a review of the state of the art, methods, advances, advantages and limitations, and future challenges of wood constructions (i.e., wood-frame, post-and-beam, mass timber and hybrid) with a particular focus on energy efficiency.

## 2. Materials and Methods

This review presents a state of the art of wood building systems and their relationship to energy efficiency. This study used the content analysis method [66,67] to select the literature to review. The main objective of the content analysis was to make accurate and valid inferences about the data collected to reveal the central aspects of previous studies. Previous studies have focused on energy efficiency improvement using by, for example, systems and insulating materials to reduce the use of heating or cooling systems—electrical energy, materials with lower EE impacts, and different building models to reduce heat loss or heat gain. Sample collection, querying, and selection were carried out in peer-reviewed articles, relevant books and technical reports as proposed by previous studies [66,68]. The keywords summarized in Table 1 were used to collect data from the ASCE Library, Engineering Village, Science Direct and Wiley Online Library databases. This search procedure for documents related to the research topics involved the following three steps. Titles, keywords and abstracts were scanned for the related keywords. The authors scaled down the search by focusing on articles, books and technical reports published from 1990 to 2021. A brief review of the abstract was conducted to filter out less relevant or unrelated literature. Documents were scanned to filter and retrieve relevant documents from the scored documents and were checked in the first round to ensure that they met the criteria. After filtering, about sixty documents with content relevant to the purpose of this paper were identified and reviewed.

Furthermore, a deeper review process was carried out using systematic queries to ensure an in-depth analysis of related articles and to confirm the conceptual depth of the review. This method followed the process proposed by Breton et al. [69]. Keywords with an asterisk (*) in Table 1 cover the central topics of the review and are not excessively restrictive. The keywords were combined in four queries using the Engineering Village database as follows:

1. ((“wood frame” AND (“building” OR “construction”)) OR “mass timber”);
2. AND (“materials” or “building materials”);
3. AND (“embodied energy” or “energy efficiency”) AND “energy”;
4. AND “efficiency”.

**Figure 1:**
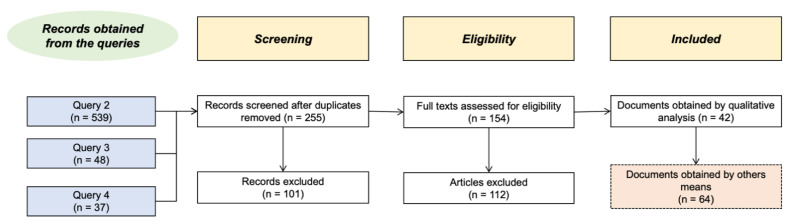
Systematic query results—adapted from preferred reporting items for systematic reviews and meta-analyses (PRISMA) flow diagram [69,70].

The resulting data extracted from the databases (i.e., Compendex, Inspec, GEOBASE, GeoRef and Knovel) for the period 1990–2021 for query 1 was 1176 records. However, after reviewing the records, it was found that most of them were not related to the main focus of this study. Therefore, queries 2 to 4 were more restrictive, leading to fewer and more relevant documents (Figure 1). For the research process, duplicate documents were removed, and titles and abstracts were screened for eligibility. Then, records were excluded if the document’s content was outside of the scope of the review (i.e., masonry, reinforced concrete or bridges, among others). Moreover, when the details of records did not provide enough information to determine whether the document was within or outside of the scope, a full review was conducted using the same criteria proposed by Breton et al. [69]. Finally, 42 articles were identified, and another 64 documents were obtained through other search strategies. The more than 100 documents selected were then reviewed to identify advances in wood buildings, particularly those focused on energy efficiency. The documents also provided background and support for other related documents to enrich the literature review.

This review is organized as follows: the first section provides an overview of the role of materials in wood buildings in improving energy performance. Subsequently, an overview of wood constructions (i.e., wood-frame, post-and-beam, mass timber and hybrid) and energy efficiency is discussed. This is followed by a brief introduction to strategies to increase the energy efficiency of constructions. Finally, a summary of future research opportunities is proposed.

## 3. Materials’ Influence on Building Energy Efficiency

Buildings’ EE suggests that the raw material extraction and product manufacturing processes have a significant impact on the building sector, especially for traditional building materials (e.g., concrete, steel, aluminum, glass, and insulation) [71,72]. The type of materials used can significantly affect a building’s EE, which will also have an impact on the building’s energy efficiency. Mithraratne and Vale [73] state that the elements of the building envelope, such as the floors, walls, roof and finishes, account for 50% of EE for a standard house. The influence of materials on the energy efficiency of the building also has been studied, taking into account high-efficiency windows, insulation materials, envelopes, ceilings and floors, thus emphasizing the EE [74].

Concerning the types of insulation materials used, for example, the American Society of Heating, Refrigerating and Air-Conditioning Engineers (ASHRAE) [75] has divided them into four groups:

1. Fibrous: This group refers to fibers that are small in size to provide air space in the material. This type of insulation is produced with organic or inorganic fibers (e.g., glass wool, rock wool, slag wool, wood wool, cellulose) that are normally bound together with an adhesive [75];
2. Granular: This group refers to nodules containing voids or hollow spaces. These materials are also considered open-cell materials due to the gases that can be transferred between the individual spaces [75];
3. Cellular: The materials in this group are made of individual cells that are either interconnected with or sealed from each other. Glass, plastics and rubber may comprise the base material, and various foaming agents are used. Cellular insulation is often further classified as either open-cell (when cells are interconnected) or closed-cell (when cells are sealed from each other) [75];
4. Reflective and treatments: This group includes materials that are added to surfaces to lower long-wave emittance, thereby reducing radiant heat transfer from the surface, such as low-emittance jackets and facing [75].

The main reason for using insulation materials in wood buildings is that they improve thermal performance since they act as a barrier, making heat gain/loss difficult. Since many of these insulators are made up of numerous microscopic air cells, they, therefore, suppress heat transfer by convention by blocking air movement. The air trapped in the material then provides increased thermal resistance [76].

Table 2 presents the thermal conductivity and EE of insulation materials. Thermal conductivity can be defined as the ability of a material to conduct heat from a high-temperature location to a low-temperature location. The smaller the thermal conductivity value, the better the insulation material [77]. Therefore, improving the thermal properties of materials can be a means to improve the thermal performance of buildings, which consequently leads to energy savings.

Since the entire construction system, not only the insulation material, impacts the energy efficiency of constructions, the thermal characteristics of structural building materials are presented in Table 3. The density and thermal conductivity of materials are the main factors contributing to thermal performance, which gives wood-based materials a clear advantage over other building materials [78]. Other properties, such as specific heat capacity and thermal diffusivity, are also important for achieving energy-efficient buildings. See [76] for the definitions of these properties.

Among the wood-based materials used in buildings, cross laminated timber (CLT), for example, provides many advantageous properties, such as high fire resistance and good thermal insulation [86]. Jayalath compared the life cycle impacts of two types of building systems, i.e., reinforced concrete (RC) and CLT for mid-rise residential buildings [87]. The results showed that the EE value of the RC buildings was 51% higher compared to the EE value of the CLT. In addition, it was also identified that this difference between the RC and CLT buildings is due to the main structural elements, such as columns, flooring, exterior walls, interior walls and roof. This is mainly due to the fact that materials, such as steel and concrete in the RC building consume much energy in its manufacturing.

As stated earlier, EE can represent as much as 60% of a building’s total energy, indicating a huge potential for energy-efficient building materials [16,17,18,19,20,21,22,23,24,78]. Dimoudia and Tompa [88] stated that the highest EE values belong to concrete and reinforcing steel, representing about 59% to 66% of the total EE of a studied building. Wood-based materials have much lower EE values compared to conventional materials, such as concrete and steel, making them a more sustainable and low-carbon construction alternative [89,90,91]. According to Buchanan and Levine, this is due to the fact that the manufacturing process for wood-based materials is much less energy-intensive than that for other construction materials. The authors also state that wood buildings have significantly lower EE compared to buildings made of other construction materials, such as brick, steel and concrete [92].

Thus, the use of wood-based materials as building elements represents significant energy savings potential by reducing EE values (see Table 3). In addition, wood-based materials and wooden buildings are less energy-intensive to produce/build and are able to absorb and store CO_2_ [112,113], contrary to concrete and steel, which require much energy to process and emit a huge amount of CO_2_ during production. Moreover, it is quite well-known that wooden buildings are lightweight, easy to build and environmentally friendly. Therefore, the choice of wood as a building element also represents an interesting strategy to improve energy efficiency in the building industry. An overview of wood constructions (i.e., wood-frame, post-and-beam, mass timber and hybrid) and energy efficiency is, therefore, discussed in the following section [65].

## 4. Wood and Building Energy Efficiency

On account of the abundance of forestry resources, wood has been used as a building material worldwide from the earliest settlements. Thanks to its versatility, this material (or its products) can be used in various applications, from a simple insulation component for constructions to a structural element in high-rise buildings. Moreover, the combination of materials provides a rigid structure that is capable of withstanding wind, earthquakes, snow and occupants and providing favorable housing conditions for the latter [114,115]. Therefore, this document presents an overview of the four main types of wood building systems: (1) wood-frame, (2) post-and-beam, (3) mass timber and (4) hybrid [116,117].

### 4.1. Wood Frame Buildings

**Figure 2:**
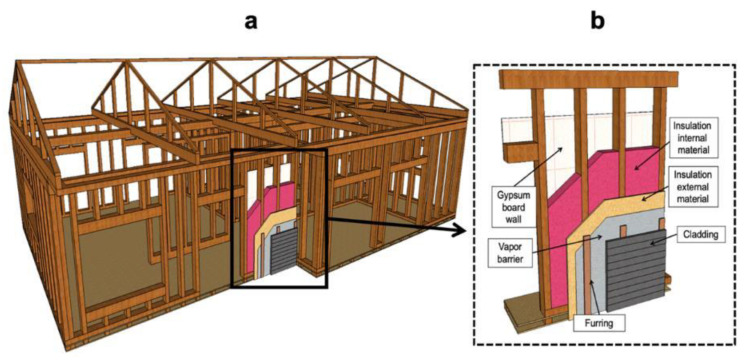
Wood frame schematic drawing: (**a**) wood-frame structure (**b**) details of wood-frame walls based on documents from Canada Mortgage and Housing Corporation [115] and the American Wood Council [119].

A wood frame combines structural and nonstructural elements. Its structural elements include wall studs, floor joists and roof trusses with floors, walls and roof sheathing. Nonstructural elements can be classified as insulation and finishing materials. Figure 2a shows a schematic drawing of the wood-frame structure, while Figure 2b shows a schematic drawing of how a wood-frame wall is built and insulated. In a wood-frame building, saw timber and wood products provide the framing system the ability to withstand vertical loads, such as snow, occupants and the building itself, as well as horizontal loads, such as earthquakes and strong winds [118]. However, it is important to emphasize that in regions where there is a risk of earthquakes and wind, it is advised to use reinforced walls, floors and roofs. Reinforcement is achieved by using a thicker sheathing board and spacing fasteners closer together [115].

When building a typical wood-frame envelope, OSB, plywood or fiber-cement board are applied as structural elements to add rigidity to the construction. They can be used on one or both sides of the walls and fastened with nails, screws or steel staples. In addition, insulation materials, such as fiberglass, cellulose, sprayed polyurethane or mineral wool (Table 2), are placed in the frame cavities as insulation material [118,120]. Studies for cold regions indicate that energy performance must take precedence when selecting materials since it impacts both heating and cooling demand [121]. Effective insulation retards heat flow through the envelope and provides a structural barrier between the internal and external environment. If well insulated, the construction stays warmer in winter and cooler in summer. Therefore, enhancing the thermal efficiency of the building results in energy savings.

From an energy efficiency point of view, the wood frame has specific mass distribution and inertia characteristics compared to, for example, masonry walls. Since it is not a homogeneous wall consisting of homogenous and continuous materials (see Figure 2), there are often concerns about the thermal efficacy of the building. Thermal bridges are normally created in buildings by repetitive structural elements and the connections between different building components. They refer to exchanges where the insulation is not homogeneous, and heat loss occurs. Thermal bridges affect the energy performance of buildings due to an increase in heat loss in winter and heat gain in summer [122]. Moreover, according to François et al. [123], two main types of thermal bridges can be found in buildings. The first is geometrical or structural, which occurs due to the shape of the building (e.g., wall/floor junctions, corners). The second is material-related and is caused by anomalies in the insulation layers. Furthermore, wood-frame buildings experience typical heat losses of 35% through walls, 15% through doors, 10% through windows, 15% through the floor and 25% through the roof [124]. A proper thermal performance-based design and appropriate insulation materials are, therefore, crucial.

For example, Chang et al. [125] investigated the EE of construction projects and found that the EE impacts of a traditional building could vary by 25% to 30% over the building’s lifetime. The EE of wood-framed and concrete-framed buildings was compared to that of a standard single detached house in the Greater Victoria area (Canada) [126]. The results showed that wood-framed constructions reduced EE by about 69% compared to the standard house. Kosny et al. [61] considered engineered wood products (plywood or OSB depending on availability) with traditional and new thermal insulators to increase the thermal performance of wood frames with double stud and truss walls. The results showed that the materials performed properly in terms of thermal resistance, i.e., higher than 3.5 m^2^K/W (U-value lower than 0.29 W/m^2^K). Furthermore, it was found that the thermal resistance of 5.3 m^2^K/W for the truss walls and double wall can be easily exceeded with thicknesses from 216 mm to 254 mm. In the case of using vacuum insulation panels (VIP) as insulators, the thermal resistance value was 9 m^2^K/W (thickness of 254 mm). It also has been found that these structures have lower thermal dispersion in the thermal bridges.

Other approaches worth mentioning refer to environmental impacts and improvements to wood-frame buildings. Liu et al. state that the construction of a typical 185 m^2^ residential house can produce 3600 kg of solid waste, approximately 900 kg, of which comes from the walls. The authors argue that there is a need for modern technologies enabling proactive design and planning for wood-frame buildings [127]. Accordingly, an interesting strategy may be the use of industrialized or prefabricated wood elements as 3D volumetric elements, i.e., modules or houses, or as prefabricated parts of a 2D panel system that are manufactured in industries under controlled conditions [128,129]. As compared to traditional on-site construction, building prefabrication comprises three main steps: (1) production of the construction components (e.g., panels and/or modules) in a controlled environment (factory), (2) delivery of the components to the site, and (3) installation of the components on-site [130]. It is interesting to note that building with prefabricated systems reduces delivery times and labor costs and that construction with controlled weather conditions is a very interesting approach for regions with severe winters [128]. Prefabrication is also a strategy to make the construction industry more sustainable by reducing waste production and demand for water, raw materials and human capital while increasing energy efficiency by reducing buildings’ thermal bridges through the use of standardized methods in a quality-controlled factory [131,132,133,134].

### 4.2. Post-and-Beam Buildings

**Figure 3:**
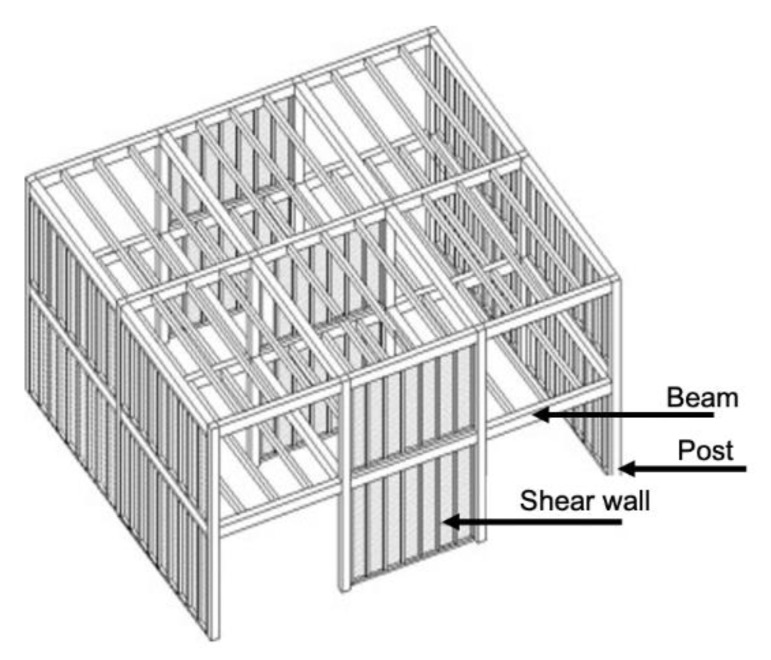
Example of a post-and-beam structure modified from [137].

According to Li et al. [135], a post-and-beam building is very similar to a wood-frame building; however, the post-and-beam structure is composed of two-dimensional assemblies: (i) horizontal components, such as floors, ceilings and roofs; and (ii) verticals components, such as walls (See Figure 3). Post-and-beam is a simple construction method in which the (traditionally rectilinear) beams support transverse purlins that are covered by a wooden deck, and the posts supporting the beams are usually arranged in a regular grid. Post-and-beam structures can be built with sawn lumber or with members made from pieces of lumber that are nailed together or to lumber, glulam, laminated veneer lumber (LVL), I-joints or poles. Plywood, OSB, lumber and CLT panels can be used for shear walls [136].

Research on the energy demand of post-and-beam buildings is still limited, and studies on airtightness, which consequently affects energy savings, are almost non-existent. However, Kim et al. [138] investigated air infiltration and various factors that reduce the energy performance of post-and-beam buildings in South Korea. The results showed that post-and-beam constructions have different airtightness performance depending on the type of openings and the degree of exposure of the shear walls. Nevertheless, the authors state that Korean post-and-beam buildings can be considered to have high airtight performance similar to those found in Europe (e.g., Finland, Norway and the UK) and the USA. Cornaro et al. [139] studied the energy efficiency potential of straw bales in a post-and-beam system. The results of the LCA analysis showed that the phase of use was responsible for about 91% of the total EE of straw walls (SW) and 85% of that of traditional walls (TW) made with bricks, thermoblock, foamed polyurethane and plaster. Moreover, using CLT panels in post-and-beam buildings provides a significant advantage in terms of energy efficiency since a separate structural timber (i.e., stud) is not required. The main reason for this is because CLT is produced with plate shapes that result in a continuous surface. Hence, air leakage and thermal bridges are significantly reduced, and the heat loss caused by thermal bridges can reach 30% [103,122].

According to Richard [140], the main advantages of post-and-beam construction include concentrated load at points, allowing for maximum planning freedom, and the fact that the structure acts as a connector to various interchangeable slabs and vertical elements. It is possible to offer continuous columns to reduce the number of joints and cantilever beams to provide additional spans. In addition, it is also recommended to use continuous posts to reduce the number and complexity of connections to be made on-site.

### 4.3. Mass Timber Buildings

**Figure 4:**
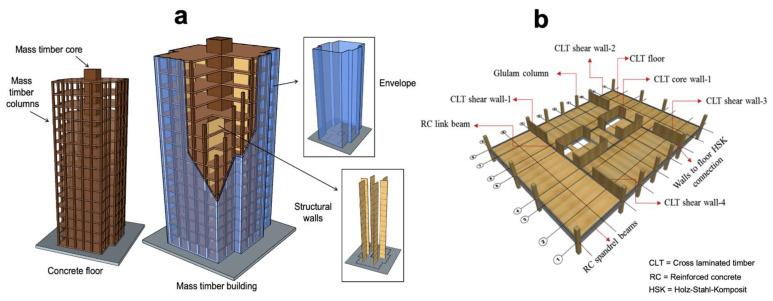
Mass timber building schematic illustration (**a**) and connections of the floor and columns (**b**) modified from [148].

In the past, 10- to 20-story buildings were built exclusively with traditional materials, such as steel and concrete. However, today, thanks to the development of CLT and glulam, it is possible to build higher with wood. Mass timber buildings are made of large-section wood products that offer the construction industry a potential alternative to steel and concrete for planar or frame elements like walls, floors, roofs and partitions, and basic building elements. These construction products and systems have attracted significant interest on account of their technical properties, cost-competitiveness and environmental impacts [141,142,143]. Concerning the international literature, mass timber buildings have been studied worldwide, and further details are available in [105,142,144,145,146]. Figure 4a presents a schematic illustration of a mass timber building, while Figure 4b provides an example of how floors and the structure (columns) are connected [147,148].

Over the past few years, several mass timber buildings have been designed with CLT panels and other wood-based materials. Traditionally, mass timber panels are fixed to the foundation or flooring diaphragm with metal connectors, such as hold-downs and angle brackets. Tests have shown that CLT panels are rigid and ductile and dissipate energy through the connections between the base and the panels, so their mechanical performance is controlled by the connections [149]. Mass timber buildings with CLT can provide potential energy savings. Guo et al. studied the energy-saving and carbon-reducing performance of CLT buildings. The results showed that buildings constructed with CLT panels outperformed RC buildings, mostly in terms of energy savings (29.4%) and reduced carbon emissions (24.6%) [150]. Simulations conducted by Setter et al. on CLT buildings in Minneapolis (USA) showed savings of 38% (USD 600) in annual heating energy, while the CLT house in Phoenix (USA) showed savings of 17% in annual cooling energy and 20% electric cooling peak savings [151]. Tettey et al. also indicate that CLT may require between 20% and 37% less energy than concrete for heating and cooling [152]. Furthermore, a 10-story modeled building studied by Khavari et al. showed that CLT provides energy savings of about USD 2090 per year compared to a light steel frame system [152].

**Figure 5:**
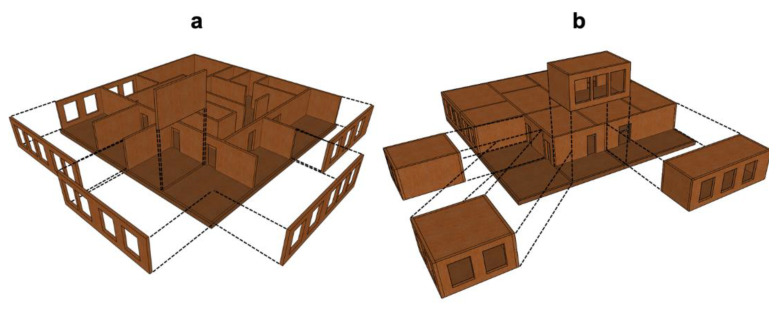
Modular mass timber systems: (**a**) planar and (**b**) volumetric.

As mentioned in previous sections, the use of prefabricated wood elements is also a very interesting strategy for mass timber buildings. Since it is possible to build with higher quality and more precision by including products, such as CLT and glulam or other custom-made products, this could lead to process innovations, such as lean manufacturing [153,154]. Using prefabricated wood elements results in (1) a reduction in on-site installation time and the overall schedule, (2) a reduction in on-site deliveries, (3) a reduction in on-site waste and related disposal costs, (4) the ability to use other compatible products and simultaneously perform off-site work under controlled conditions, (5) a reduction in the number of change orders issued and requests for information or improvements, and (6) a reduction in the scheduling phase and on-site labor costs for follow-up trades [155]. Although this is an extremely important topic, as Kedir and Hall [154] have pointed out, studies focusing on energy efficiency and new forms of construction for mass timber buildings are still limited. Industrialized components can be used in mass timber buildings as planar components (Figure 5a), i.e., walls, floors or ceiling slabs, and as volumetric components corresponding to room modules (Figure 5b), i.e., semi-independent units [145].

In studying the growth of 2D and 3D methods of industrialized construction in North America in new and existing enterprises, Pullen et al. [156] found that companies building larger structures generally use more stable or rigid materials. In other words, the authors state that between timber, steel and other types of material (concrete or unique lightweight plastics), timber dominates the low-rise market, while steel has the advantage for high-rises. On the other hand, it is not surprising that CLT and glulam are most often used for mid-rise buildings (4- to 6-stories) and typically not used for higher buildings (over 14 stories) [134,156,157].

The expansion of research and proven case studies involving taller CLT-based and glulam structures may change this trend. For example, the moisture conditions of mass timber products for an 8-story building were studied in Portland (USA) over one year by monitoring the moisture content of wood products in different building phases, including panel transport, building assembly, enclosure of the building and in situ drying [142]. The results showed that mass timber buildings built during the rainy season presented high moisture levels in wood products.

William Perkin High School in Greenford (UK) is a four-story timber complex of 3860 m^3^ built using a combination of CLT and glulam for the structure and architectural components. It was originally supposed to have a concrete frame, and changing from a concrete frame to a CLT and glulam structure reduced the embedded carbon of the superstructure by about 1500 tons of CO_2_ [158]. Another advantage of building with CLT is how quickly it can be erected. Dalston Works, a 10-story building in the UK, was built in 18 months and using CLT as the building material reduced on-site deliveries by almost 80% compared to a conventional site of concrete due to a better understanding of and more confidence in the engineered wood product [159].

### 4.4. Hybrid Buildings

**Figure 6:**
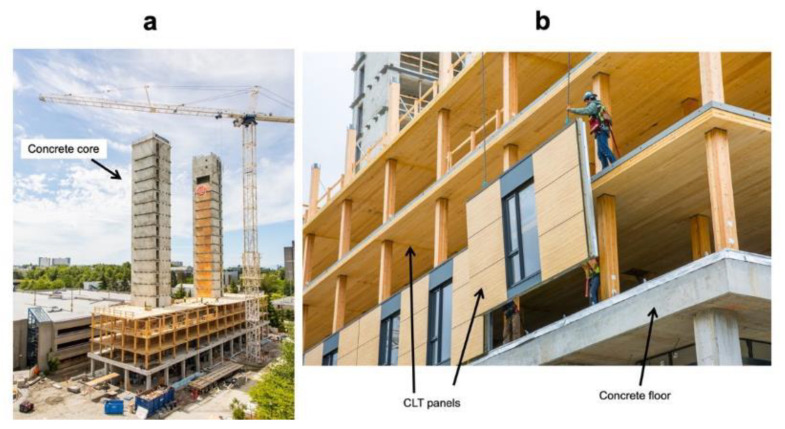
Brock Commons building progress: (**a**) concrete core and initial floor overview and (**b**) envelope cross laminated timber (CLT) panel installation. Modified from naturallywood.com; photographer: K.K. Law.

In hybrid building systems, mass timber elements, such as CLT and glulam, are combined with traditional building materials, such as steel and concrete [160]. Hybrid buildings have lower carbon emissions, a faster construction period and a lighter structural system than traditional reinforced concrete buildings [161]. In addition, components are strategically combined with increasing the height of wood buildings by improving the load-bearing capacity, as is pointed out in some studies [162,163,164]. Hybrid building systems can be used for elements (hybrid slabs/diaphragms, hybrid beams, hybrid columns, hybrid diagonals, hybrid shear walls) and/or building system levels (hybrid shear wall system, tube system, vertical mixed system), as shown in Figure 6 [163,165].

Some examples of hybrid buildings that have been built around the world include Treet and Mjøstårnet in Norway, Forté in Australia, and Brock Commons Tallwood House and Origine in Canada [166,167,168,169,170,171,172,173]. In taller buildings, the envelope mitigates the external forces acting on the building and helps to maintain comfortable thermal, visual and acoustic conditions. In addition, the envelope is an essential element of a building because it not only serves as an esthetic element but also resists the main load-bearing structure [174]. Despite their considerable benefits, mass timber and hybrid buildings are still facing several challenges, especially those related to costs [156,175]. The material is more expensive compared to traditional construction materials (concrete and steel), as has been argued in previous sections [156]. In addition, when panels are not installed properly, considerable acoustic issues may result [176]. Furthermore, as is indicated by Ahmed and Arocho [177], in the case of the USA, a lack of design codes and specifications makes it more difficult for many developers to use mass timber materials (CLT). The authors also discuss the fact that there is a limited number of firms that manufacture mass timber elements in the U.S., making the delivery of materials impractical and the cost of transportation high.

Another challenge for both mass timber and hybrid constructions is related to the sensitivity of wood products to moisture. Wood is a hygroscopic natural material that is prone to degrade significantly, from minor swelling to complete loss of structural strength due to fungi attack, when it remains moist for a long time. It is, therefore, important to take into account when designing with timber that this material must remain protected from high moisture levels during its structural lifetime, especially for heavily loaded components of mass timber and hybrid buildings [142,178]. Moreover, as is discussed in a recent study by Voulpiotis et al. [178], wood products are about 5 times less dense than RC and 15 times less dense than structural steel. The primary benefit of a lighter building composed of smaller foundations becomes a shortcoming because it becomes much more sensitive to critical lateral loads as it increases in height.

It is also important to highlight that, as an emerging technology, little attention has been paid to date to the environmental and energy impacts of hybrid buildings. However, recent research by Li et al. [179] investigated the potential benefits and limitations of using a hybrid system in Australia through simulation and parametric assessment. The results showed that wood-based materials, such as CLT, LVL, OSB had lower EE impacts. Furthermore, according to Reddy [180], the correct use of materials in buildings is crucial to achieving low-carbon and low-embodied energy constructions. The author also mentions that the use of alternative energy-efficient building technologies leads to a reduction in EE of about 50%. Using products such as CLT and glulam can be an efficient approach to increase the energy efficiency of wood buildings.

Since hybrid systems, like previously mentioned CLT systems, do not require a separate structural timber (stud), hybrid buildings have fewer thermal bridges and improved thermal efficiency [103]. Moreover, Pierobon et al. [160] have shown that hybrid CLT buildings save about 8% of non-renewable (fossil-based) energy compared to RC buildings. Robertson et al. [181] investigated and compared the environmental impacts of two building systems—a traditional cast-in-place (i.e., reinforced concrete frame) system and a hybrid system using CLT and glulam. The cradle-to-gate analysis showed that for the hybrid system, construction energy ranged from 6% to 14% of total EE, while with cast-in-place concrete, the energy range was considerably higher, at 15% to 25%.

In addition to the facts about wooden buildings mentioned in the previous sections, consideration should be given to using strategies to improve the energy efficiency of these construction systems. With this in mind, some strategies, including materials, techniques and concepts that can be adopted in wood building systems to improve energy efficiency, are presented and discussed in the following section.

## 5. Strategies to Improve Building Energy Efficiency

**Figure 7:**
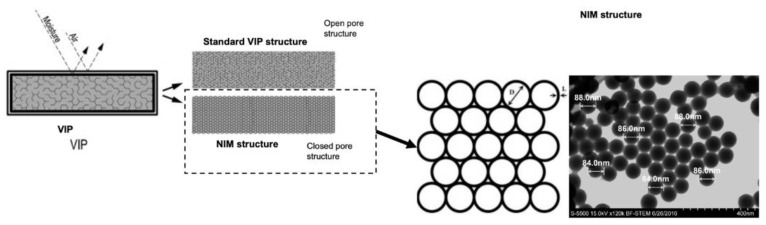
Comparison of the structure of a vacuum insulation panel (VIP) and nano-insulation material (NIM). Source: modified from [217,218,219].

Given the increasing demand for energy in buildings, strategies must be considered to reduce energy use for cooling and heating constructions, taking into account the fact that EE is responsible for more than 60% of total energy use over the life cycle of a traditional building [182]. For this reason, the International Energy Agency (IEA) and Aslani et al. [35] have proposed some technologies, which are reviewed in this study, to improve the energy efficiency of building components (e.g., ceilings, doors, external walls, floors, roof coverings and windows) over their lifetime:

1. *Insulation materials:* As mentioned in previous sections, for wood building systems, such as wood-frame, post-and-beam, mass timber and hybrid constructions, insulators played an important role in reducing thermal losses and keeping buildings heated in winter and cooled in summer. A wood-frame envelope (building) with appropriate insulation can provide an environment that is 5°C warmer in winter and 10°C cooler in summer [35]. Many types of insulation materials are available, including ones made of inorganic materials, such as ceramic materials, glass wool, rock wool and slag wool, and ones made of organic materials, including cane, cellulose, cotton, kenaf and wood particles, among others [183] (see Table 3). XI et al. [184] developed a binderless insulator board using kenaf fibers that have thermal conductivity properties similar to those of traditional insulation material (rock wool). Zhou et al. developed cotton stalk insulation boards (without binders) that are potential candidates to replace perlite and vermiculite insulators [185]. In North America, for example, the insulation materials most commonly used for wood-frame and post-and-beam buildings are glass wool, followed by expanded polystyrene (EPS), which accounts for 44.3% and 23.5% by volume, respectively [186,187].
2. *Reflective surfaces:* This strategy makes the building (façade) capable of reflecting sunlight. Thus, infrared, visible, and ultraviolet light are all important when considering reflective surfaces. Reflective surfaces are an interesting strategy to improve the energy efficiency of all types of wood building systems. A study carried out in the United States on residential and commercial buildings showed that the surface temperature of buildings could be reduced by about 10 °C by using this strategy [188]. In addition, the use of reflective surfaces on wood-frame buildings results in energy savings of 4% to 9% (4% to 6% in cold climates) [189].
3. *Building airtightness:* this approach plays a critical role for the buildings for energy-efficiency buildings as the energy performance can be significantly reduced by poor airtightness [190]. Such a topic has aroused in the 1970s but still continues as an important strategy. According to Cooper et al. [191], many researchers have pointed out that proper airtightness is a requirement for buildings’ energy efficiency since the consumption caused by unintended building air leakage can account for 13–50% and 4–20% of the overall heating and cooling demand, respectively [192,193,194,195].
4. *Cool roofs:* These roofs are used for radiation heat transfer, providing space for cooling in the buildings. It reduces surface temperature by reflecting more solar radiation into the sky comparing to conventional roofs and consequently reduces heat flow from the roof to the building. These roofs could be recommended to reduce building air conditioning loads for wooden buildings. According to Dehwah and Krarti [196], for the wood-frame constructions, cool roofs reduced annual energy use for space cooling by about 44% and that for space heating by up to 17%. For warm regions, the use of cool roofs has been shown to reduce peak demand and cooling energy by 10% to 30% [197]. According to Boixo et al. [198], cool roofs in Andalucía (Spain) can provide energy savings of about 295,000 kWh per year, which represents 2% of overall residential electricity demand for flat-roofed buildings. Moreover, these savings avoid 136,000 tons of CO_2_ per year from being produced from electricity production.
5. *Green roofs:* These are referring to totally or partly green spaces covering buildings. Green roofs are systems that make plants grow in the roof. This type of roof prevents heat from entering the building using water evaporation while protecting the roof from sunlight and energy loss [35]. According to Coma et al. [59], green roofs could reduce building energy consumption by about 16.7% in warm regions. A green roof reduced the flow of heat by 70% to 90% in summer and by 10% to 30% in winter compared to a traditional roof [199]. Green roofs are also an interesting strategy to protect wood structures from igniting [200,201].
6. *Glazed windows:* This type of window refers to the glass panes incorporated in a window frame (also called an insulating glass unit or IGU). In this system, the air sealed in between the panes acts as an insulating layer [35]. A large number of techniques can be used in all types of wood buildings to improve the thermal efficiency of IGUs, such as the use of coated glass [58], multi-layer glass [202], vacuum glass [203] or smart glass [204], and incorporating materials (e.g., gas or aerogel) in the cavity between the panes of glass [57]. Fasi et al. showed that double-paned clear-glass windows could annually reduce lighting, cooling and total energy consumption by 70%, 8% and 14%, respectively [205].
7. *Window shade:* This strategy involves using a window shade to prevent direct sun exposure inside the building either continuously or at specific times of day [35]. Tzempelikos et al. [206] stated that this method could reduce the secondary (lighting, heating and cooling) energy consumption of a building in Montreal (Canada) by 31%. Liu et al. studied the use of shading devices on opaque facades to reduce energy demand. This work was carried out in near-extreme summer conditions using energy simulations of typical buildings in Hong Kong [207]. The results showed that with an optimal configuration, the highest energy savings for the smallest total area of shade panels were observed at different tilt angles. The findings also showed potential energy savings of more than 8% for shading panels used on flats with west-facing façades. Given this scope, the use of window shading could be an approach to consider for low- and mid-rise (wood-frame and post-and-beam) buildings.
8. *Low-conductivity window frames:* Materials such as extruded vinyl, glass fiber reinforced polyester (GFRP), polyvinyl chloride (PVC) and unplasticized polyvinyl chloride (uPVC) are used to produce the frame. Low-conductivity window frames can reduce heat loss by 25% to 40% for typical building assemblies. Windows with polyurethane, urethane, glass wool and vermiculite flakes in the window frame cavity have also been studied. It has been found that the lower the thermal conductivity of the insulation, the lower the heat transfer coefficient, consequently, the greater the thermal efficiency of the window system. Furthermore, a new strategy used in wood-frame building systems in Canada [50] is to fill window frame cavities with aerogels (having a thermal conductivity of about 0.02 W/mK), which results in a reduction in heat transfer of up to 29% for IGUs.
9. *Building information modeling (BIM):* Berardi and Jafarpur [3] state that improvements in the shape, envelope and operating systems of buildings represent the largest research opportunity to reduce the energy consumption of future constructions in North America. According to Won and Cheng [208], the implementation of building information modeling (BIM) is a very promising approach to overcome this challenge, especially for wood buildings. BIM is used to develop a digital representation of a building’s components. By allowing users to extract geometric information from the project, these data can be used to manage and improve the technical aspects of the building before its construction phase. Furthermore, from an energy savings perspective, using Autodesk Revit BIM software (Version 2018, Autodesk, Inc., United States ) and an energy assessment tool FirstRate5 (Nationwide House Energy Rating Scheme, Australia) is a potential solution to systematically study variations in energy use during the operational phase considering geographic location, climatic conditions, shape, form and material variations. On the other hand, as indicated in a very recent study by Tushar et al. [209], little attention is paid to energy savings and environmental impacts concurrently with these design parameters. Moreover, as indicated in [209], energy data from the operational phase is important for all building processes to assess the influence of its corresponding EE.
10. *Phase changing materials (PCMs):* Improving the energy efficiency of buildings through energy storage is a very interesting approach for a less energy-consuming electricity system. Consequently, the use of PCMs is one way to regulate indoor temperature by shifting the peak load to off-peak hours and reducing the need for heating and cooling energy [210]. PCMs can store a large amount of latent heat by undergoing a phase change (typically from solid to liquid), which adds thermal mass to the building envelope and thus reduces the energy demand of wood building envelopes [211]. The literature shows that the use of PCMs in well-insulated residential buildings can reduce energy use for heating or cooling by up to 25% [212,213,214]. Gypsum plasterboard with incorporated PCMs (capric and stearic acid) was studied by Sari et al. [215]. The authors argued that the plasterboard absorbed 25 wt% of the PCM and showed no leakage signs after 5000 thermal cycling tests (melting/freezing cycling). In addition, the plasterboard’s thermal performance improved, reducing the indoor temperature by 1.3 °C. Mathis et al. [49] investigated MDF panels with plastic and PCMs, and HDF on top to enclose the PCM pouches. For this experiment, the following PCM mixtures were used: a blend of capric and lauric acids and two commercial products (PureTemp^®^20 and PureTemp^®^23 (PureTemp, Park Glen Rd, MN, United States). The results revealed that the latent panel was stable, making the materials suitable for building applications. The panel made with Puretemp^®^23 embodied much energy, up to 57.1 Jg^−1^ with a melting point of 22.2 °C. These results show that the use of PCMs in the envelope of wood building systems could be an interesting strategy to improve the energy efficiency of buildings.
11. *Nano-insulation materials (NIMs):* Another strategy to improve the energy performance of wood buildings is the use of nanotechnology-based insulation materials or NIMs. These materials are homogeneous composites consisting of open or closed nanopore particles in the range of 0.1 nm–100 nm in size with low thermal conductivity (less than 0.04 W/mK) in perfect conditions [216]. According to Gao et al., NIMs have a thermal conductivity of about 0.02 W/mK on account of the predominantly size-dependent thermal conduction that occurs at the nanometer scale. Figure 7 compares the structure of VIPs and NIMs. Although it is mentioned in previous sections, it is important to also stress here that not all types of strategies reviewed have been the subject of the same amount of research and development efforts and available literature. There is a vast amount of literature on topics dealing with green roofs, organic and inorganic PCMs and insulation materials, whereas other areas, such as window shades and NIMs, have gaps in the literature and provide very interesting research opportunities for future studies.

## 6. Concluding Remarks and Future Research Opportunities

**Figure 8:**
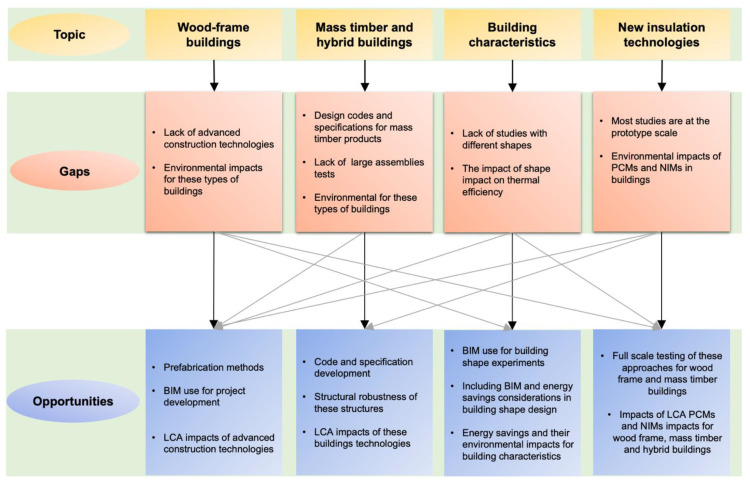
Research opportunities for wood constructions.

This section presents some recommendations for developing more energy-efficient wood buildings and other aspects identified in the literature to improve the overall performance of wooden buildings. What has been done in the area of construction, materials and technologies to improve the total energy efficiency of wood building systems is summarized in the previous sections. Future research directions can be defined based on the research gaps in the main research topics. Four major topics and their gaps and opportunities are highlighted in Figure 8. In addition, despite some points being directly related, it is important to note that indirect studies also present opportunities for future research.

1. The literature has shown that although on-site mid-rise construction has well-established methods and products in the industry, there is a lack of research into the use of advanced construction technologies. Usage examples include incorporating prefabricated systems in wood-frame and post-and-beam buildings and emphasizing the potential environmental benefits (energy savings, GHG emission reductions, etc.) that these technologies might bring to the industry. Given the fact that most of the studies found focus particularly on materials and not on the building system as a whole, including BIM during pre-project development, taking energy savings into account could be an area for consideration. Moreover, the literature review identified that in post-and-beam studies, limited research has been done on energy use.
2. Mass timber and hybrid systems offer innovative solutions for the construction industry. From an energy efficiency perspective, it was found that CLT can achieve energy savings of about 40% compared to traditional building systems such as concrete and light steel frame. It was also found that although hybrid buildings are an emerging technology to date, little attention has been given to their environmental and energy impacts. Furthermore, although not directly related to the energy efficiency of construction, an important point that was identified by this review and deserves to be highlighted is the lack of development of design codes and specifications for the use of mass timber materials. Those that focus on well-established concepts for concrete and steel structures, such as structural robustness—which, according to the Voulpiotis et al. [178], is still not well comprehended because of the complexity of wood properties and the challenge of testing large assemblies—therefore, represent a research opportunity to be seized. In addition, as is the case for wood-frame structures, studies of the potential environmental benefits of mass timber and hybrid systems as a whole are still a research gap.
3. Choosing the most appropriate building shape and correct orientation could reduce energy consumption by 30–40% [220]. However, to date, studies concentrating on such topics and the energy savings of wood buildings generally overlook architectural variability and architectural features related to the functional needs of the building. Therefore, improving the envelope, operating systems and shape of wood-frame buildings, especially in North America, still represents the largest opportunity to reduce building energy consumption [3].
4. Phase changing materials (PCMs) and nano-insulating materials (NIMs) represent energy-saving potential for wood buildings. However, it was found that most of the studies pertaining to them were based on prototype elements and that there was little practical application of these technologies. Thus, full-scale testing, with practical application, is a noteworthy field to explore, especially using such technologies in wood-frame and mass timber buildings. In addition, practical cases must be considered to evaluate the thermal and energy performance and life cycle analysis (LCA) of using such elements.

## Figures and Tables

**Table 1:** Primary and secondary keywords used for the search.

Primary Keyword	Buildings *	Materials *	Energy *
Secondary keyword	Wood buildings	Wood	Embodied energy *
	Wood frame *	Bio-based	Energy efficiency *
	Mass timber *	Building materials *	Energy improvement
	Constructions *	Sustainable materials	

* Keywords used in the systematic queries.

**Table 2:** Thermal characteristics and embodied energy of insulation materials.

Form	Material	Density (kg/m^3^)	Thermal Conductivity (W/mK)	Typical Applications	Embodied Energy (MJ/kg)	References
Sprayed-in-place	Cellulose	24–36	0.054–0.046	Attic retrofitting, frame sidewalls.	0.94–3.30	[76,79,80]
Foamed-in-place	Polyurethane	40–55	0.024–0.023	Roofs, cavities, irregular and rough surfaces.	72.10–102.10	
Blankets: batts or rolls	Fiberglass	12–56	0.040–0.030	Walls, ceilings, partitions, prefabricated houses, irregular surfaces.	11.00–41.81	[76,79,81,82]
	Rock wool	40–200	0.037–0.040		11.30–16.80	
	Polyethylene	35–40	0.041–0.029	Ceilings, hangers, wrapping, carpet underlay, expansion joints.	51.00–103.00	
Poured-in	Fiberglass	10–48	0.038–0.030	Cavities, around obstructions.	11.00–41.81	[76,79,83,84]
	Rock wool	—	0.042–0.040	Cavities.	11.30–16.80	
	Cellulose	24–36	0.054–0.046	Small cavities.	0.94–3.30	
	Perlite	32–176	0.060–0.040	Fill or mixed with Portland cement.	0.66–10.00	
	Vermiculite	64–130	0.068–0.063	Ceilings, cavity walls, cores of hollow-core blocks.	0.72–7.20	
Board	Fiberglass	24–112	0.035–0.032	Cavity walls, roofs, prefabricated houses.	11.00–41.81	[76,79,80,85]
	Expanded polystyrene	16–35	0.038–0.037	Walls, roofs, floors, basements, foundations. Must be covered inside and outside (fire and weather protection).	58.40–151.00	
	Extruded polystyrene	26–45	0.032–0.030		58.40–151.00	
	Polyurethane	40–55	0.024–0.023		65.20–110.00	
	Vacuum Insulation Panels (VIP)	—	0.004–0.003	Walls, roofs, floors, perimeter, basements, foundations.	—	
Reflective systems	Aluminized thin sheets	—	Reduces only radiant heat transfer	Ceilings, walls, floors. Most effective to reduce downward heat flow.	115.00–157.10	[76,79]

**Table 3:** Thermal characteristics and embodied energy of commonly used building materials.

Material	Density (kg/m^3^)	Thermal Conductivity (W/m°C)	Typical Applications	Embodied Energy (MJ/kg)	References
Timber—softwood	450	0.12–0.14	Studs, trimmers, cripplers, other structural elements of wood frames	0.30–13.00	[93,94,95,96,97,98,99]
Timber—hardwood	700	0.17–0.23		7.00–18.00	
Oriented strand boards (OSB)	650	0.13–0.24	Sub-flooring, single-layer flooring, wall and roof sheathing, ceilings/decks, structural insulated panels, webs for wood i-joists, industrial containers, mezzanines	10.00–15.00	[93,94,95,97,98,100,101,102]
Hardboard	1000	0.12–0.29		16.00–35.00	
Particleboard	600	0.12–0.17		4.00–15.00	
Medium density fiberboards (MDF)	600	0.011–0.14		8.90–11.00	
Plywood	700	0.12–0.15		10.00–20.00	
Cross laminated timber (CLT)	485	0.13–0.10	Floors, walls, roofing	4.90–10.00	[93,94,95,96,97,98,103,104,105,106]
Glulam	600	0.12–0.13	Beams, columns	8.00–14.00	
Gypsum board	900	0.25–0.80	Heavy-wear locations where durability and resistance to abrasions are required	3.48–6.75	[93,97,98,107,108,109]
Cement-bonded board	1200	0.23–0.80	Sub-flooring, single-layer flooring, walls, ceiling/deck sheathing	4.80–6.75	[93,97,98]
Concrete	1600	0.40–0.57	Sub-flooring, beams, columns	1.70–23.90	[93,97,98,107,110,111]
Steel	7850	50.00–64.00	—	25.00–45.68	

## Data Availability

Data sharing is not applicable to this article.
